# Patient Perceptions of COVID-19 Precautions and Their Effects on Experiences With Hand Surgery

**DOI:** 10.1016/j.jhsg.2021.04.003

**Published:** 2021-05-12

**Authors:** Amr M. Tawfik, Jeremy M. Silver, Brian M. Katt, Aneesh Patankar, Michael Rivlin, Pedro K. Beredjiklian

**Affiliations:** ∗ Rothman Orthopaedic Institute, Philadelphia, PA; † Rutgers Robert Wood Johnson Medical School, New Brunswick, NJ

**Keywords:** COVID-19, Elective surgery, Orthopedic surgery, Patient safety, Upper extremity

## Abstract

**Purpose:**

The purpose of this study is to evaluate patient perceptions of COVID-19 precautions and how these precautions have affected their hand and upper extremity surgery experience.

**Methods:**

We sent an 18-item survey to 1,213 patients who underwent elective hand and upper extremity surgery at 1 academic institution from October 2020 to January 2021. The survey consisted of questions related to patient demographics, treatment delays due to COVID-19, and patient perceptions of COVID-19 precautions. Descriptive statistics were performed to analyze the survey responses. Responses for patients aged 18–50 and 51+ were compared using a chi-square analysis for categorical variables and a Student *t*-test for continuous variables.

**Results:**

Out of 1,213 invitations, 384 survey respondents completed the survey (31.6%). Of the respondents, 16.8% reported delaying medical treatment for an average of 123.2 days because of COVID-19. The preventative measures were found to be adequate by 95% of patients. Only 2.6% of patients reported experiencing surgical delays due to preoperative COVID-19 testing or other COVID-19-related precautions. COVID-19 testing was seen as necessary by 88% of patients, and 74% did not find COVID-19 testing to be a barrier to their surgery. Patients aged 51+ were more likely to delay seeking medical treatment than younger patients (19.3% vs 9.1%, respectively). Furthermore, those that did delay seeking treatment waited longer on average than their younger counterparts (136.1 vs 72.9 days, respectively).

**Conclusions:**

In conclusion, patients undergoing hand and upper extremity surgery typically do not find COVID-19 precautions to be a significant barrier to care and understand their importance. Despite this, many patients, particularly older ones, are delaying medical care for extended periods of time. It is important for hand surgeons to acknowledge their patients’ perspectives and work to educate patients on evolving surgical safety guidelines.

**Clinical relevance:**

Patient perspectives of current COVID-19 precautions can help inform hand surgeons on areas for continued patient education.

As the COVID-19 pandemic began to impact the United States in March 2020, the health care system was forced to reevaluate the dissemination of resources. Ultimately, widespread recommendations were made to limit nonemergent and elective surgeries.^1^ As the pandemic has progressed, elective surgeries have resumed, with a wide variety of testing protocols and guidelines in place to minimize the spread of the coronavirus.^2^, ^3^, ^4^, ^5^, ^6^ Key tenets of these guidelines are to prioritize patient safety while also limiting the economic burden stemming from the widespread cancellation of elective surgeries.^7^^,^^8^ The burden of these cancellations is especially considerable in orthopedic surgery, with an estimated backlog of more than 1 million orthopedic cases.^9^ For this reason, there has been an extensive push to safely resume elective orthopedic surgery.^10^^,^^11^

Despite protocol implementation, it is unclear how patients view these precautions or whether they impact surgical experience and patient care. Several studies in other specialties have explored the patient perspective on resuming elective procedures during the pandemic, with mixed results.^12^, ^13^, ^14^, ^15^ There are also several orthopedic-specific studies that have examined patient perspectives, with most of them focusing on total joint arthroplasty.^16^, ^17^, ^18^, ^19^ These studies have shown varying impacts on patient well-being, progression of disease, and anxiety surrounding elective procedures in the COVID-19 era. It remains unclear, however, how these protocols alter the patient experience for hand and upper extremity surgery. The purpose of this study is to evaluate patient perceptions of COVID-19 precautions and how these precautions have affected the hand and upper extremity surgery experience.

## Materials and Methods

This study was designed as a descriptive study at a single institution. Between October 2020 and January 2021, all patients at the Rothman Orthopaedic Institute, Philadelphia, Pennsylvania, who were 18 years or older and who underwent elective hand and upper extremity surgery at our institution were sent an invitation to complete an 18-item anonymous survey. For our study, we defined elective surgery as any nonemergent surgery. Patients who were scheduled for a surgical procedure at a future date by 1 of 13 hand and upper extremity surgeons at our institution were identified in our hospital system database. On the first Tuesday following their procedure, patients were emailed a single invitation to complete the anonymous survey. The survey consisted of questions related to patient demographics, treatment delays due to COVID-19, and patient perceptions of COVID-19 precautions. The full contents of the survey can be seen in Appendix 1 (available online on the *Journal’s* website at www.jhsgo.org). COVID-19 precautions at our institution consisted of: (1) mandatory masks worn at all times by all staff, patients, and visitors, with masks provided at the entrance if needed; (2) temperature checks and screening questions for everyone entering the surgical center; (3) additional cleaning and disinfection of the entire facility; (4) patient family/friends/visitors required to wait outside of the building unless necessary to deliver safe care (e.g., parents of a minor child or person with disabilities requiring special assistance); and (5) patients were required to have a negative COVID-19 test taken 48 to 72 hours prior to surgery, followed by self-quarantine.

For comparative analysis, patient responses were stratified into age groups 18–50 and 51 and older. This stratification was chosen based on data demonstrating that patients older than 50 had a greater infection fatality ratio due to COVID-19.^20^ Continuous variables were reported as means and standard deviations, and categorical variables were reported as counts and percentages of the total group. A Student *t*-test and a chi-square test were used for statistical analyses of continuous and categorical variables, respectively. Significance was established at a *P* value of < .05.

## Results

The survey was completed by 384 respondents out of 1,213 total invitations (31.6% response rate). We excluded 32 responses due to incomplete surveys, leaving 352 surveys to be used in the final analysis.

Most respondents (75.0%) were 51 years or older; of the total cohort, 47.4% were men and 52.6% women. The most common self-reported medical problems were obesity (18.2%) and diabetes (10.2%). For 43.5% of respondents, the upper extremity issue had lasted over 6 months, and 47.2% reported that their issue was due to trauma (Table 1).

**Table 1:** Survey Respondent Demographic Data

Variable	n (%)∗
Sex	
Male	167 (47.44)
Female	185 (52.56)
Age	
18–30	29 (8.24)
31–50	59 (16.76)
51–70	194 (55.11)
71–90	69 (19.60)
>90	1 (0.28)
Medical problems	
Cancer	34 (9.66)
Chronic kidney disease	3 (0.85)
COPD	12 (3.41)
Obesity (BMI > 30 kg/m^2^)	64 (18.18)
Heart conditions	25 (7.10)
Type 2 diabetes	36 (10.23)
How long have you had symptoms for your upper extremity problem?	
1 day to 1 week	31 (8.81)
1 week to 1 month	93 (26.42)
1 month to 6 months	75 (21.31)
>6 months	153 (43.47)
Did your upper extremity problem result from recent trauma or accident?	
Yes	166 (47.16)
No	186 (52.84)

BMI, body mass index; COPD, chronic obstructive pulmonary disease.

∗ n = 352.

In total, 59 patients (17.8%) reported delaying treatment for their upper extremity issue due to COVID-19, for an average of 123 days. When asked whether screening for COVID-19 in the operating room was adequate, 65% agreed or strongly agreed. The COVID-19 test caused no discomfort for 63.9% of patients and mild discomfort for 26.4%. Surgery was rarely delayed (2.6%) due to the process of obtaining COVID-19 testing (Table 2).

**Table 2:** Effects on Patient Care Due to COVID-19 and its Precautions

Variable	n (%)∗
Did you delay seeking medical attention for your upper extremity problem because of the COVID-19 pandemic?	
Yes	59 (16.76)
No	293 (83.24)
How many days did you delay treatment? Mean ± SD	123.24 ± 51.27
During your office visit, do you feel that preventative measures, specifically against COVID-19, were adequate visit?	
Yes	334 (94.89)
No	18 (5.11)
Screening for COVID-19 in the operating room was adequate	
Strongly agree	103 (29.26)
Agree	126 (35.80)
Neutral	103 (29.26)
Disagree	13 (3.69)
Strongly disagree	7 (1.99)
Please rate the level of discomfort of the COVID-19 test	
No discomfort	225 (63.92)
Mild discomfort	93 (26.42)
Moderate discomfort	31 (8.81)
Significant discomfort	3 (0.85)
Was your surgery delayed because of COVID-19 testing?	
Yes	9 (2.56)
No	343 (97.44)

∗ n = 352.

Most (88.1%) patients agreed that COVID-19 testing prior to surgery is necessary, although 11.9% of patients disagreed with or were neutral about the importance of COVID-19 testing prior to surgery. COVID-19 testing was seen as a barrier to surgery by 8.8% of patients, and 16.8% were neutral. Patients typically felt safer in the operating room knowing that all patients were tested prior to surgery (86.1%). Despite this, patients had mixed responses when asked whether they should quarantine prior to surgery following a negative COVID-19 test. As shown in Table 3, 22.4% of patients disapproved of family and friends not being allowed to accompany them into the surgical facility.

**Table 3:** Patient Perceptions of COVID-19 Precautions

Statement, n (%)	Strongly Agree	Agree	Neutral	Disagree	Strongly Disagree
COVID-19 testing prior to surgery is necessary	225 (63.92)	85 (24.15)	26 (7.39)	8 (2.27)	8 (2.27)
COVID-19 testing prior to surgery is inconvenient	11 (3.13)	51 (14.49)	76 (21.59)	84 (23.86)	130 (36.93)
COVID-19 testing was a barrier to surgery	11 (3.13)	20 (5.68)	59 (16.76)	89 (25.28)	173 (49.15)
I feel safer in the operating room knowing all patients are tested prior to surgery	207 (58.81)	96 (27.27)	32 (9.09)	11 (3.13)	6 (1.70)
Quarantining after COVID-19 testing and prior to surgery is necessary	123 (34.94)	112 (31.82)	76 (21.59)	27 (7.67)	14 (3.98)
If you have a negative COVID-19 test, you should not have to change your behavior before surgery	19 (5.40)	44 (12.50)	78 (22.16)	98 (27.84)	113 (32.10)
I did not care that my family member/friend/significant other was not allowed to come to the operating room with me	90 (25.57)	131 (37.22)	52 (14.77)	49 (13.92)	30 (8.52)

A comparison of the responses for patients aged 18 to 50 and those 51 and older are summarized in Table 4. Patients 51 years and older were significantly more likely to delay seeking treatment due to COVID-19 as compared to younger patients (19.3% vs 9.1%, respectively; *P* = .010). Furthermore, when patients did delay seeking treatment, older patients delayed for significantly longer than younger patients (136 vs 73 days, respectively; *P* < .001).

**Table 4:** Delaying Treatment in Younger Patients Versus Older Patients

Variable, n (%)	Patients 18–50 Years Old	Patients 51+ Years Old	*P* Value
Subjects, n	88	264	
Sex			.951
Male	42 (47.73)	125 (47.35)	
Female	46 (52.27)	139 (52.65)	
Duration of upper extremity issue			.008∗
1 day to 1 week	7 (7.95)	24 (9.09)	
1 week to 1 month	39 (44.32)	54 (20.45)	
1 month to 6 months	17 (19.32)	58 (21.97)	
>6 months	25 (28.41)	128 (48.48)	
Did you delay seeking medical treatment due to COVID-19			.010∗
Yes	8 (9.09)	51 (19.32)	
No	80 (90.91)	213 (80.68)	
How many days did you delay treatment? Mean ± SD	72.92 ± 41.27	136.09 ± 45.59	<.001∗

∗ Significant at ≤ .05.

## Discussion

The purpose of this study was to evaluate patient perceptions of COVID-19 precautions and how these precautions affected the hand and upper extremity surgery experience. Of note, most of the patients who responded to the survey were over 50 years old. We believe that this cohort represents the perceptions of the average orthopedic hand surgery patient, as prior studies have found that surgical patients, specifically hand surgery patients, are well above the median age of the general population (55 vs 39 years old, respectively).^21^, ^22^, ^23^

In our cohort, a number of patients were older than 50 and had medical conditions associated with increased COVID-19 severity, including cancer, obesity, and diabetes.^20^^,^^24^ A study by Chang et al^16^ found that patients with higher COVID-19 risk scores were less willing to pursue surgery during COVID-19. Therefore, it is unsurprising that 18% of patients in our study reported delaying care due to COVID-19, particularly those that were older than 50. Messaging in the United States media has highlighted that COVID-19 can be particularly deadly for those older than 50 or those with comorbidities.^25^, ^26^, ^27^ This has likely discouraged these populations from seeking medical care, as hospitals are viewed to be COVID-19 hot spots.^18^ However, recent data suggest that the implementation of preoperative COVID-19 screening and self-quarantining for elective orthopedic surgery has not been associated with any cases of COVID-19 as confirmed by polymerase chain reaction testing.^28^

It is imperative for physicians and the general media to communicate that COVID-19 transmission is relatively rare when COVID-19 precautions are implemented.^28^, ^29^, ^30^ This messaging is vital, as delays in elective surgery are not without consequence. In our study, patients were delaying surgery for an average of 123 days, and 43% had their issue for over 6 months. Physicians should emphasize that elective surgery is not simply optional surgery, as even minor delays in these procedures are associated with increased risks of a surgical site infection and longer recovery time.^31^ Beyond surgical complications, delays can lead to a decreased quality of life and an increased likelihood of depression, particularly for chronic conditions.^17^^,^^32^^,^^33^ When planning surgical interventions, physicians should discuss with patients the chronicity of the issue, assess its impact on their quality of life, and alleviate apprehension regarding COVID-19 risks in order to allow patients to reach an informed decision.

The findings of our study underscore the need for the medical community to address false perceptions regarding COVID-19 safety precautions. While most patients understood the value of testing prior to surgery, 12% were neutral or disagreed about its necessity. Most concerning were the discordant views surrounding the significance of preoperative quarantining. The value of quarantining prior to elective surgery must be stressed to patients, as it relies on patient compliance and is vital to minimizing COVID-19 transmission.^28^ This is supported by a study conducted during the severe acute respiratory syndrome outbreak, which found that people who understood the importance of quarantining were more likely to comply with regulations.^34^ Furthermore, a recent Gallop poll found that hospitals and health agencies were the most trusted source for COVID-19-related information.^35^ Health care providers should identify patients with COVID-19 misconceptions and capitalize on this established trust to correct them.

Although our study only reported that a minority of patients found COVID-19 testing inconvenient and a barrier to surgery, it is imperative to address these sentiments. Our institution required a negative COVID-19 test 48–72 hours prior to surgery, and even though delays in surgery due to COVID-19 testing were rare, the limitations of this practice should still be recognized. When agreeing to schedule surgery, patients may need several days off work for preoperative COVID-19 testing and the subsequent quarantine. Further, if a patient cannot comply with testing regulations, their surgical case is cancelled, with limited ability to schedule another patient in their place. While this testing window is necessary to reduce the risk of COVID-19 exposure in surgical centers, physicians should recognize the inconvenience it can present and accommodate surgical scheduling when possible.

The limitation of visitors can place a tremendous emotional toll on patients and families as they navigate health challenges.^36^ In our study, 77.5% of our respondents did not care or were neutral about the no-visitor policy implemented at our institution. These data are comparable to those in a Howard et al^18^ study that showed 84.2% of patients undergoing elective orthopedic surgery were willing to comply with strict self-isolation for 2 weeks before and after the procedure and understood the no-visitor policy when it was explained. These results again demonstrate the willingness of patients to comply with precautions when they are adequately educated on the rationale.

We are aware of a few limitations associated with our study. Our study only captured patients that ultimately decided to present for elective surgery, potentially underrepresenting the percentage of patients that chose to delay intervention. Delays in intervention may have been further underrepresented by our choice of a definition for elective surgery. Patients who had nonemergent fractures, such as wrist fractures, may have felt that they had no choice but to present for care. Additionally, the study had a relatively low response rate (31.6%) and may have been subject to recall bias, as some of the questions required recollection of the patient’s upper extremity condition. Finally, this study was done through 1 division at a single institution in the United States roughly 7 to 10 months into the pandemic. Protocols implemented at our institution at that time may have differed compared to those at other global institutions.

In conclusion, hand and upper extremity surgery patients typically do not find COVID-19 precautions a significant barrier to care, and understand their importance. Despite this, many patients are delaying medical care for extended periods of time, particularly older patients. It is important for hand surgeons to acknowledge their patients’ perspectives and work to educate patients on evolving surgical safety guidelines.

## References

[1] Centers for Medicare and Medicaid Services. *Non-emergent, elective medical services, and treatment recommendations**.* Available at: https://www.hhs.gov/guidance/document/non-emergent-elective-medical-services-and-treatment-recommendations. Published August 31, 2020, Accessed February 15, 2021.

[2] Centers for Medicare and Medicaid Services. *Centers for Medicare & Medicaid Services (CMS) recommendations re-opening facilities to provide non-emergent non-COVID-19 healthcare*. Available at: https://www.cms.gov/newsroom/press-releases/cms-issues-recommendations-re-open-health-care-systems-areas-low-incidence-covid-19. Published April 19, 2020, Accessed February 16, 2021.

[3] Al-Omar K., Bakkar S., Khasawneh L., Donatini G., Miccoli P. (2020). Resuming elective surgery in the time of COVID-19: a safe and comprehensive strategy. Updates Surg.

[4] Diaz A., Sarac B.A., Schoenbrunner A.R., Janis J.E., Pawlik T.M. (2020). Elective surgery in the time of COVID-19. Am J Surg.

[5] Schlosser M., Signorelli H., Gregg W., Korwek K., Sands K. (2021). COVID-19 testing processes and patient protections for resumption of elective surgery. Am J Surg.

[6] Kaye K., Paprottka F., Escudero R. (2020). Elective, non-urgent procedures and aesthetic surgery in the wake of SARS-COVID-19: considerations regarding safety, feasibility and impact on clinical management. Aesthetic Plast Surg.

[7] O’Connor C.M., Anoushiravani A.A., DiCaprio M.R., Healy W.L., Iorio R. (2020). Economic recovery after the COVID-19 pandemic: resuming elective orthopedic surgery and total joint arthroplasty. J Arthroplasty.

[8] Lockey S.D., Nelson P.C., Kessler M.J., Kessler M.W. (2021). Approaching “elective” surgery in the era of COVID-19. J Hand Surg Am.

[9] Jain A., Jain P., Aggarwal S. (2020). SARS-CoV-2 impact on elective orthopaedic surgery: implications for post-pandemic recovery. J Bone Joint Surg Am.

[10] Ding B.T.K., Tan K.G., Oh J.Y.-L., Lee K.T. (2020). Orthopaedic surgery after COVID-19–a blueprint for resuming elective surgery after a pandemic. Int J Surg.

[11] Iyengar K.P., Jain V.K., Vaish A., Vaishya R., Maini L., Lal H. (2020). Post COVID-19: planning strategies to resume orthopaedic surgery -challenges and considerations. J Clin Orthop Trauma.

[12] Moverman M.A., Puzzitiello R.N., Pagani N.R., Barnes C.L., Jawa A., Menendez M.E. (2021). Public perceptions of resuming elective surgery during the COVID-19 pandemic. J Arthroplasty.

[13] Campi R., Tellini R., Grosso A.A. (2021). Deferring elective urologic surgery during the COVID-19 pandemic: the patients’ perspective. Urology.

[14] Sii S.S.Z., Chean C.S., Sandland-Taylor L.E. (2020). Impact of COVID-19 on cataract surgery- patients’ perceptions while waiting for cataract surgery and their willingness to attend hospital for cataract surgery during the easing of lockdown period. Eye (Lond).

[15] Nicholson P., Ali F.R., Patalay R., Craythorne E., Mallipeddi R. (2021). Patient perceptions of Mohs micrographic surgery during the COVID-19 pandemic and lessons for the next outbreak. Clin Exp Dermatol.

[16] Chang J., Wignadasan W., Kontoghiorghe C. (2020). Restarting elective orthopaedic services during the COVID-19 pandemic. Bone Jt Open.

[17] Wilson J.M., Schwartz A.M., Grissom H.E. ( 2020). Patient perceptions of COVID-19-related surgical delay: an analysis of patients awaiting total hip and knee arthroplasty. HSS J.

[18] Howard A., Hodgson H., Vun J. (2021). The patient’s perspective on returning to elective surgery after COVID-19. Ann Surg.

[19] Morris J.A., Super J., Huntley D., Ashdown T., Harland W., Anakwe R. (2020). Waiting lists for symptomatic joint arthritis are not benign: prioritizing patients for surgery in the setting of COVID-19. Bone Jt Open.

[20] Perez-Saez J., Lauer S.A., Kaiser L. (2021). Serology-informed estimates of SARS-CoV-2 infection fatality risk in Geneva, Switzerland. Lancet Infect Dis.

[21] Fowler A.J., Abbott T.E.F., Prowle J., Pearse R.M. (2019). Age of patients undergoing surgery. Br J Surg.

[22] Naran S., Imbriglia J.E., Bilonick R.A., Taieb A., Wollstein R. (2010). A demographic analysis of cubital tunnel syndrome. Ann Plast Surg.

[23] Bahar-Moni A.S., Abdullah S., Fauzi H., Chee-Yuen S.Y., Abdul-Razak F.Z., Sapuan J. (2019). Demographics of patients undergoing carpal tunnel release in an urban tertiary hospital in Malaysia. Malays Orthop J.

[24] CDC Certain medical conditions and risk for severe COVID-19 illness. https://www.cdc.gov/coronavirus/2019-ncov/need-extra-precautions/people-with-medical-conditions.html.

[25] ScienceDaily COVID-19 is dangerous for middle-aged adults, not just the elderly: study examines infection fatality rates for COVID-19. https://www.sciencedaily.com/releases/2021/01/210121131806.htm.

[26] AARP 95 percent of Americans killed by COVID were 50+. https://www.aarp.org/health/conditions-treatments/info-2020/coronavirus-deaths-older-adults.html.

[27] CDC Older adults and COVID-19. https://www.cdc.gov/coronavirus/2019-ncov/need-extra-precautions/older-adults.html.

[28] Nishitani K., Nagao M., Matsuda S. (2021). Self-quarantine programme and pre-operative SARS-CoV-2 PCR screening for orthopaedic elective surgery: experience from Japan. Int Orthop.

[29] Kader N., Clement N.D., Patel V.R., Caplan N., Banaszkiewicz P., Kader D. (2020). The theoretical mortality risk of an asymptomatic patient with a negative SARS-CoV-2 test developing COVID-19 following elective orthopaedic surgery. Bone Joint J.

[30] Gammeri E., Cillo G.M., Sunthareswaran R., Magro T. (2020). Is a “COVID-19-free” hospital the answer to resuming elective surgery during the current pandemic? Results from the first available prospective study. Surgery.

[31] Vogel T.R., Dombrovskiy V.Y., Lowry S.F. (2010). In-hospital delay of elective surgery for high volume procedures: the impact on infectious complications. J Am Coll Surg.

[32] Fishbain D.A., Cutler R., Rosomoff H.L., Rosomoff R.S. (1997). Chronic pain-associated depression: antecedent or consequence of chronic pain? A review. Clin J Pain.

[33] Leite A.A., Costa A.J.G., de Lima B.A.M., Padilha A.V.L., de Albuquerque E.C., Marques C.D.L. (2011). Comorbidities in patients with osteoarthritis: frequency and impact on pain and physical function. Rev Bras Reumatol.

[34] Frank Newport Six emerging conclusions: public opinion and COVID-19. https://news.gallup.com/opinion/polling-matters/304646/five-emerging-conclusions-public-opinion-covid.aspx.

[35] Reynolds D.L., Garay J.R., Deamond S.L., Moran M.K., Gold W., Styra R. (2008). Understanding, compliance and psychological impact of the SARS quarantine experience. Epidemiol Infect.

[36] Siddiqi H. (2020). To suffer alone: hospital visitation policies during COVID-19. J Hosp Med.

