# Supplementary material for: Patient Perceptions of COVID-19 Precautions and Their Effects on Experiences With Hand Surgery
Source: J Hand Surg Glob Online. 2021 May 12;3(4):167–71. doi: 10.1016/j.jhsg.2021.04.003 (PMC8113162; doi:10.1016/j.jhsg.2021.04.003)
Supplement: Appendix 1 [file mmc1.pdf]

1. Sex

- ◯ Male
- ◯ Female

2. Please select your age group

- ◯ 18-30
- ◯ 31-50
- ◯ 51-70
- ◯ 70-90
- ◯ Over 91

3. Please indicate if you have a history of the following medical problems:

- ☐ Cancer
- ☐ Chronic Kidney Disease
- ☐ Chronic Obstructive Pulmonary Disease (COPD)
- ☐ Obesity (BMI > 30)
- ☐ Heart Conditions (such as Heart Failure, Coronary Artery Disease, abnormal stress test, history of stents of bypass, or Cardiomyopathy)
- ☐ Type 2 Diabetes

4. How long have you had symptoms for your upper extremity problem?

- ◯ 1 day to 1 week
- ◯ 1 week to 1 month
- ◯ 1 month to 6 months
- ◯ > 6 months

5. Did your upper extremity problem result from recent trauma or accident?

- ◯ Yes
- ◯ No

6. Did you delay seeking medical attention for your upper extremity problem because of the COVID-19 pandemic?

- Yes
- No

7. How many days did you delay treatment?

0 100 200

8. During your office visit, do you feel that preventative measures, specifically against COVID, were adequate?

- Yes
- No

9. Please rate your perception of the following statement: Screening for COVID in the operating room was adequate

Strongly Agree	Agree	Neutral	Disagree	Strongly Disagree

10. Please rate the level of discomfort of the COVID Test

No Discomfort	Mild Discomfort	Moderate Discomfort	Significant Discomfort	Severe Discomfort

11. Was your surgery delayed because of COVID testing? (ie not getting your results in time for the procedure)

- Yes
- No

12. Please rate your perception of the following:

	Strongly Agree	Agree	Neutral	Disagree	Strongly Disagree
COVID testing prior to surgery is necessary	◯	◯	◯	◯	◯
COVID testing prior to surgery is inconvenient	◯	◯	◯	◯	◯
COVID testing was a barrier to surgery	◯	◯	◯	◯	◯
I feel safer in an operating room knowing all patients are tested prior to surgery	◯	◯	◯	◯	◯
Quarantining after COVID testing and prior to surgery is necessary	◯	◯	◯	◯	◯
If you have a negative COVID test, you should not have to change your behavior before surgery	◯	◯	◯	◯	◯
I did not care that my family member/friend/significant other was not allowed to come to the operating room with me	◯	◯	◯	◯	◯
